# Five-year outcomes of HAART at a non-governmental treatment center in Lima, Peru

**DOI:** 10.1186/1758-2652-13-S4-P173

**Published:** 2010-11-08

**Authors:** N Castillo, C Agurto, C Benites, JA Hidalgo

**Affiliations:** 1 Via Libre, Lima, Peru

## Background

Ministry of Health in Peru launched an HIV treatment program with the initial support of the Global Fund in 2004. A few months later, our institution, an NGO working in HIV prevention, entered the treatment program in order to contribute to the national efforts to improve access to HIV care. There are no other available reports on long-term results of HAART in our country.

## Methods

Retrospective medical records review of 149 consecutive patients that initiated HIV treatment through the national program at our outpatient Clinic between February and July 2005. Main clinical and laboratory outcomes were recorded and reported.

## Results

After an observation period of five years, 127 (85.2%) persons continue their care at our Clinic. Mean baseline age was 34.6 yrs. The group included 34 women (22.8%). Eight patients died (5.3%), 7 (4.7%) patients were referred to other centers at their request, and 7 (4.7%) were lost to follow up. Eighty-five patients (57.0%) were treatment naive at the moment of joining our treatment program. One hundred sixteen patients show current virological success (HIV VL<400 c/ml), with an ITT success rate of 77.8%, and an "on treatment" success rate of 89.7%. Ninety-five persons have shown sustained virological success over the 5 years (89 on NNRTI-based regimens). Mean baseline CD4 cell count at the moment of treatment initiation was 86/µL. Mean CD4 cell count at five years follow up is 254/µL. Forty-five patients have required change of a drug due to an adverse event (most commonly during the initial months of therapy). Causes of mortality were opportunistic infections related to IRIS or previously active (TB, PCP, disseminated histoplasmosis) and 2 cases of NHL. All deaths occurred within then initial year of treatment. Significant morbidities such as cancer or TB after the first year of treatment occurred in 8 patients (5.3%). No cardiovascular event has been observed in this cohort so far.

## Conclusions

Outcomes observed at 5 years of HAART are comparable to the ones observed in other treatment programs developed in limited resource settings. The model of care supported by our NGO is highly successful with no additional support from public funding other than medications and monitoring laboratories.

